# Synergistic Effects of A Combined Treatment of Glioblastoma U251 Cells with An Anti-miR-10b-5p Molecule and An AntiCancer Agent Based on 1-(3′,4′,5′-Trimethoxyphenyl)-2-Aryl-1*H*-Imidazole Scaffold

**DOI:** 10.3390/ijms23115991

**Published:** 2022-05-26

**Authors:** Matteo Zurlo, Romeo Romagnoli, Paola Oliva, Jessica Gasparello, Alessia Finotti, Roberto Gambari

**Affiliations:** 1 Department of Life Sciences and Biotechnology, Ferrara University, 44121 Ferrara, Italy; matteo.zurlo@unife.it (M.Z.); jessica.gasparello@unife.it (J.G.); 2 Department of Chemical, Pharmaceutical and Agricultural Sciences, Ferrara University, 44121 Ferrara, Italy; romeo.romagnoli@unife.it (R.R.); paola.oliva@unife.it (P.O.)

**Keywords:** microRNA, anti-miR, miR-10b-5p, glioma, apoptosis, tubulin, 1-(3′,4′,5′-trimethoxyphenyl)-2-aryl-1*H*-imidazole, combination therapy

## Abstract

(1) Background: In the development of new and more effective anticancer approaches, combined treatments appear of great interest. Combination therapy could be of importance in the management of glioblastoma (GBM), a lethal malignancy that accounts for 42% of cancer of the central nervous system, with a median survival of 15 months. This study aimed to verify the activity on a glioblastoma cancer cell line of one of the most active compounds of a novel series of tubulin polymerization inhibitors based on the 1-(3′,4′,5′-trimethoxyphenyl)-2-aryl-1*H*-imidazole scaffold, used in combination with a miRNA inhibitor molecule targeting the oncomiRNA miR-10b-5p. This microRNA was selected in consideration of the role of miR-10b-5p on the onset and progression of glioblastoma. (2) Methods: Apoptosis was analyzed by Annexin-V and Caspase 3/7 assays, efficacy of the anti-miR-10b-5p was assessed by determining the miR-10b-5p content by RT-qPCR. (3) Results: The results obtained show that a “combination therapy” performed by combining the use of an anti-miR-10b-5p and a 1-(3′,4′,5′-trimethoxyphenyl)-2-aryl-1*H*-imidazole derivative is an encouraging strategy to boost the efficacy of anticancer therapies and at the same time to reduce side effects.

## 1. Introduction

Glioblastoma (GBM) is a lethal malignant tumor accounting for 42% of the tumors of the central nervous system, with the median survival being 15 months [1,2,3,4]. Currently, there is no effective pharmacological treatment available, and the first-line drug used, Temozolomide (TMZ), is, on average, able to prolong the life expectancy of treated patients by only a few months [4]. Additionally, many forms of glioblastoma are or become resistant to TMZ over time [4,5,6]. Therefore, there is an urgent need to find new drugs, therapeutic approaches, and protocols (such as combined therapy) to develop anti-glioma therapies more effective than those currently available, especially on TMZ-resistant tumors.

Combined treatment might be of great interest in order to develop effective therapeutic protocols for glioblastoma [7,8,9,10,11,12]. In this respect, we have recently reported a possible combined therapeutic approach for GBM based on the use of microRNA inhibitors and anti-GBM molecules. For example, we have found that sulforaphane (SFN) and a peptide-nucleic acid (PNA) targeting microRNA miR-15b-5p synergistically act by inducing apoptosis of the glioblastoma U251 cells [13]. Therefore, the PNA-a15b might be proposed in “combo-therapy” associated with SFN. Overall, this study suggests the feasibility of using combined treatments in which chemical bioactive agents directed against selected tumor-associated pathways might be administered together with antisense biomolecules targeting oncomiRNAs [14,15,16,17,18,19].

Targeting microRNAs is appealing, as these short non-coding RNA sequences act as key gene regulators by repressing translation or causing the cleavage of the RNA transcripts they target [20,21,22]. There is now a large consensus on the fact that the altered expression of miRNAs may be involved in the pathogenesis of cancer [23,24,25]. In particular, those miRNAs that are upregulated in cancer and cause down-regulation of target tumor suppressor mRNAs are defined as “oncomiRNAs” and “metastamiRNAs” [25]. Concerning GBM, a possible microRNA target is miR-10b-5p [26,27,28,29,30,31]. There is, in fact, strong evidence that miR-10b-5p is overexpressed in malignant glioma and associated with tumor invasive factors [26,27,29] and GBM aggressiveness [31]. Importantly, miR-10b-5p is not expressed (or expressed at very low levels) in non-cancerous brain tissues [27]. In agreement with the concept that miR-10b-5p represents a unique therapeutic target for GBM, El Fatimy et al. reported a very interesting study based on the CRISPR-Cas9 gene editing [32]. The results of this study were focused on the effects of miR-10b gene editing on growth of cultured human glioma cells, tumor-initiating stem-like cells, and mouse GBM xenografts, as well as the oncogene-induced transformation of normal astrocytes. Interestingly, miR-10b-5p gene ablation was found to be lethal for glioma cell cultures and established intracranial tumors [32]. In agreement with this information, miR-10b-5p has been selected as a molecular target for pharmaceutical treatment based on the antagomiRNA approach [33,34,35,36].

A possible pathway to be targeted in combined therapy of GBM is that controlling tubulin polymerization [37,38,39]. In this respect, Bordji et al., 2014 reported that several studies have indicated aberrant levels of βIII-tubulin (βIII-t) in human GBM. βIII-t overexpression was linked to increasing malignancy in glial tumors and described to determine the onset of resistance to chemotherapy [40]. Accordingly, tubulin polymerization has been suggested as a biochemical target for chemotherapy of GBM [41,42,43,44,45,46,47].

In this respect, some of us have recently published a study focusing on the development and characterization of a novel series of tubulin polymerization inhibitors based on the 1-(3′,4′,5′-trimethoxyphenyl)-2-aryl-1*H*-imidazole scaffold and designed as *cis*-restricted combretastatin A-4 (CA-4) analogs [48]. A chloro and ethoxy group at the *meta*- and *para*-positions, respectively, produced the most active compound in the series (**4o**), with IC_50_ values of 0.4–3.8 nM against a panel of seven different cancer cell lines. Experiments carried out in a syngeneic mouse model demonstrated high antitumor activity of **4o**, which significantly reduced the tumor mass [48].

The aim of the present study was to verify the activity of compound **4o** on a glioma cell line when used in combination with a commercial miRNA inhibitor molecule targeting the oncomiRNA miR-10b-5p (antimiR-10b-5p). The possible synergistic action of **4o** and antimiR-10b-5p was studied by evaluation of the effects on cell viability and apoptosis, as it is already known that tubulin polymerization inhibition, as well as miR-10b-5p downregulation, are able to induce apoptosis in glioma cells [33,48,49].

## 2. Results

### 2.1. Effects of Compound ***4o*** on U251 Cell Growth and Apoptosis

**Figure 1:**
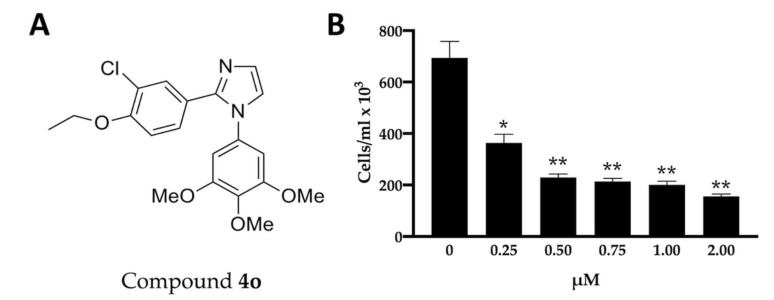
Structure of compound **4o** and its effects on U251 cell proliferation. (**A**). Structure of compound **4o**. (**B**). Effects of derivative **4o** on U251 cell proliferation. U251 cells were cultured for 3 days in the absence or in the presence of the indicated concentrations of compound **4o**. Results represent the cell number/mL values (mean ± SD; *n* = 3). *p* < 0.05 (*, significant), *p* < 0.01 (**; highly significant).

The chemical structure of 2-(3′-chloro-4′-ethoxyphenyl)-1-(3′,4′,5′-trimethoxyphenyl)-1*H*-imidazole (**4o**) is shown in Figure 1A. Figure 1B shows the effects of **4o** on cell proliferation of U251 cells. After 72 h of cell culture employing the indicated experimental conditions, the cell number/mL was determined. The data indicate that inhibition of U251 cell growth by **4o** reaches the maximum values when 0.5–2 μΜ concentrations of **4o** are used. This finding is in line with the experimental data reported by Romagnoli et al., indicating that **4o** exert antiproliferative effects on different tumor cell lines, including glioblastoma [48].

**Figure 2:**
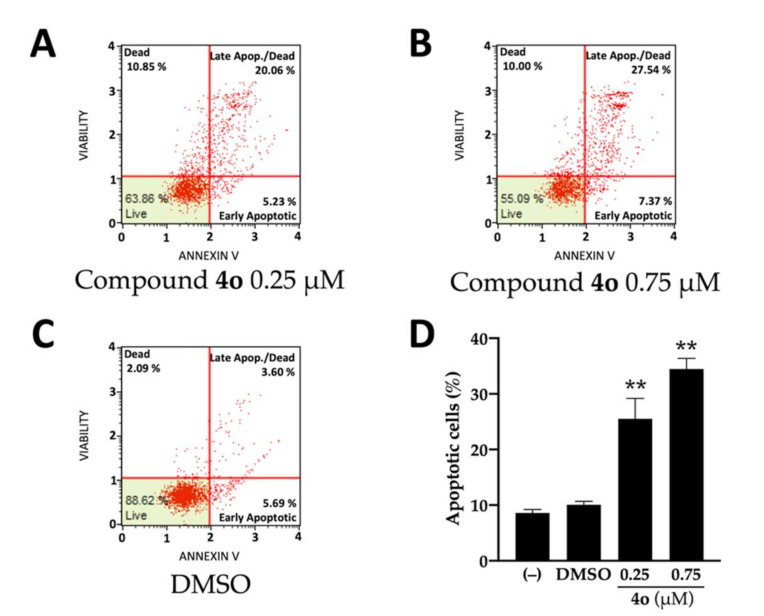
Effects of compound **4o** on apoptosis. The annexin V assay was performed on U251 cells treated for 3 days in the presence of the indicated concentrations of **4o** (**A**) and (**B**). (**C**). Vehicle treated U251 cells. (**D**). Summary representing the mean increase of % of apoptotic cells ± SD (*n* = 4). *p* < 0.01 (**; highly significant).

The compound **4o** is also able to induce apoptosis of U251 cells, as depicted in Figure 2. In fact, compound **4o** at 0.25 μM concentration is sufficient for generating a sharp increase in total apoptotic cells (in the case of late apoptosis, the % increases from 3.60% in control untreated cells to 20.06% and 27.54%, in U251 cells cultured in the presence of 0.25 and 0.75 μM compound **4o**, respectively). The 0.25 μM concentration of **4o** was selected to limit the expected side-effects of high dosages of **4o** on treated cells. The analysis of the effects of compound **4o** administered at concentrations ranging from 50 nM to 2 μM is reported in Supplementary Material (Figures S1 and S2), showing again a concentration-dependent effect on induction of apoptosis.

### 2.2. Effects of Anti-miR-10b-5p on U251 Apoptosis

**Figure 3:**
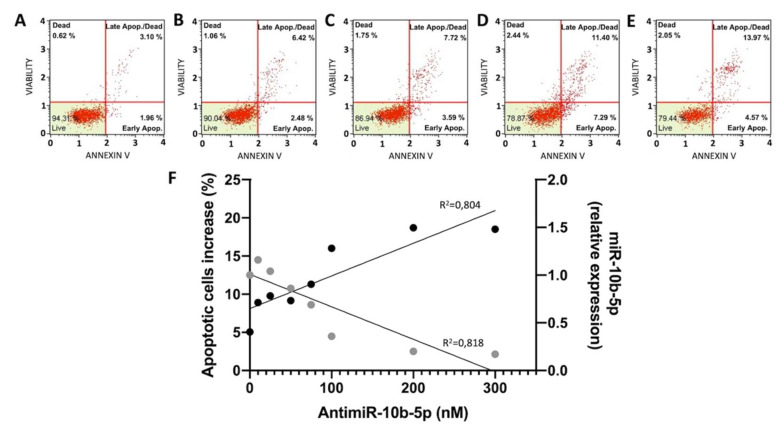
Effects of anti-miR-10b-5p on apoptosis. (**A**–**E**). Representative Annexin V assay plots performed on untreated U251 cells (−)or cells cultured for 3 days in the presence of DMSO or the indicated concentrations of anti-miR-10b-5p (C. 50 nM, D. 200 nM, E. 300 nM). The complete set of data is presented in Supplementary Material Figures S3 and S4. (**F**). Summary representing the increase of % of apoptotic cells (black dots) related to the decrease of miR-10b-5p following treatment with the anti-miR-10b-5p molecule (grey dots), R squared values obtained by linear regression test are presented on the plot.

The effects of antimiR-10b-5p on apoptosis are shown in Figure 3 and in Supplementary Material Figures S3 and S4. In Figure 3A–E, representative plots are shown, demonstrating that the used anti-miR-10b-5p molecule induces apoptosis (Figure 3C–E), while only minor effects of the vehicle (lipofectamine RNAiMAX) were appreciable (Figure 3B). Figure 3F shows the relationship between administered concentrations of anti-miR-10b-5p and apoptosis. Interestingly, the increase of induced apoptosis is associated with a decrease in the expression of miR-10b-5p following treatment with the anti-miRNA molecule, as verified by the Pearson correlation test (*p* = 0.0007). The complete set of the results generating the summary data presented in Figure 3F is included in the Supplementary Material Figures S3 and S4.

The treatment with the anti-miR-10b-5p molecules is expected to have only limited effects on “normal” brain cell lines and/or tissues, as this microRNA is not expressed (or expressed at very low levels) in non-cancerous brain tissues [27,36,50]. While future experiments analyzing the effects of the anti-miR-10b-5p on normal “healthy” brain cell lines appear to be of relevance, we wanted to further analyze the specificity of the effects on microRNAs by exploring the effects of the treatment on miRNAs demonstrated to be onco-suppressor in brain cells, such as miR-101-3p [51], miR-424-5p [52], and miR-93-5p [53]. These data are presented in Supplementary Materials Figure S5 and clearly indicate that while inhibition of miR-10b-5p was as expected confirmed (fully in agreement with Figure 3F), no inhibitory effects were found when miR-101-3p, miR-424-5p, and miR-93-5p were considered, further supporting the hypothesis that the treatment is highly specific.

### 2.3. Effects of Compound ***4o*** on miR-10b-5p Expression and Combined Effects with Anti-miR-10b-5p Transfection

**Figure 4:**
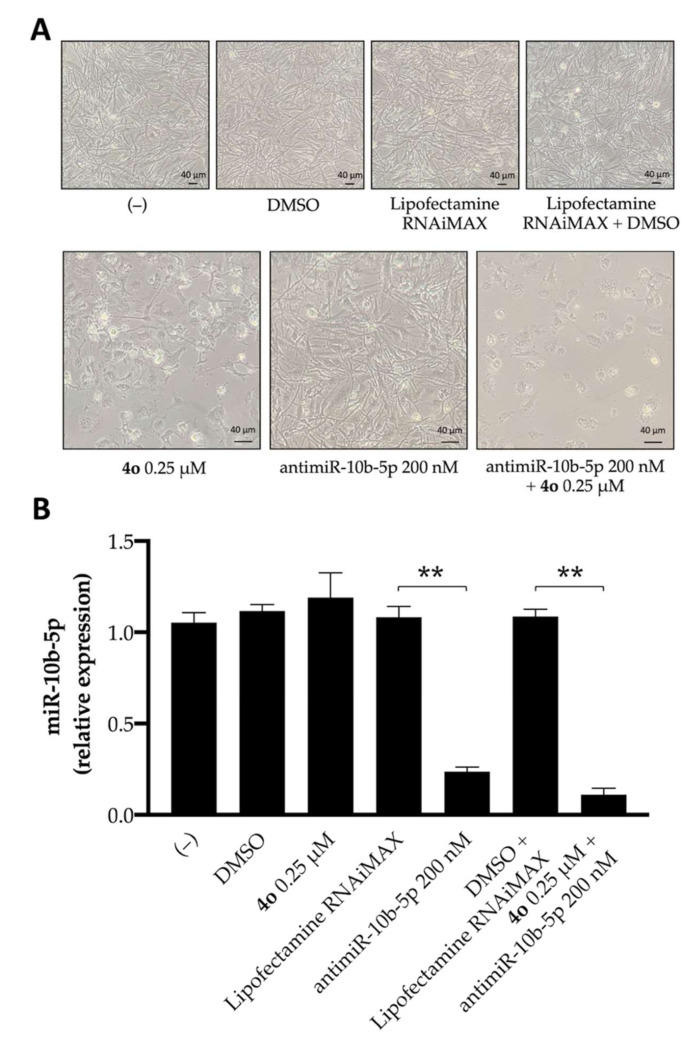
Effects of compound **4o** and antimiR-10b-5p on U251 morphology and expression of miR-10b-5p. (**A**). Studies focusing on U251 morphology. (**B**). Effects on miR-10b-5p, analyzed by RT-qPCR. Cells were either untreated (−) or treated as indicated for 3 days. In panel B, the results represent miR-10b-5p expression with respect to relatives Lipofectamine and Lipofectamine plus DMSO controls (mean ± SD; *n* = 3). *p* < 0.01 (**; highly significant).

Figure 4 shows that compound **4o** has only minor effects on the expression of miR-10b. This experiment was conducted by exposing for 72 h human glioma U251 cells to DMSO (the vehicle of **4o**), lipofectamine RNAiMAX (the vehicle for antimiR-10b-5p), **4o**, antimiR-10b-5p, and a combination of **4o** and antimiR-10b-5p (with the relative DMSO + lipofectamine control). After this period of cell culture, the cells were analyzed for morphology (Figure 4A) and miR-10b-5p expression (Figure 4B). To this end, RNA was isolated and miR-10b-5p sequences were quantified by RT-qPCR. Interestingly, no effects on cell growth and morphology were appreciable in control untreated cells, as well as in U251 cells cultured with the DMSO and lipofectamine RNAiMAX vehicles, even when they were used in combination. By contrast, alterations in morphology and cell growth efficiency were observed following treatment with **4o**, anti-miR-10b-5p, or the combined treatment using these molecules (Figure 4A). This finding is fully in agreement with the MTT assay included in Supplementary Material Figure S6.

Concerning the RT-qPCR data (Figure 4B), the antimiR-10b-5p causes strong inhibition of miR-10b-5p expression. DMSO and lipofectamine were not effective, and compound **4o** did not change significantly miR-10b-5p expression. The combined treatment was the most effective in inhibiting expression of miR-10b-5p.

### 2.4. Co-Treatment of U251 Cells with Compound ***4o*** and AntimiR-10b-5p: Effects on Cell Cycle

**Figure 5:**
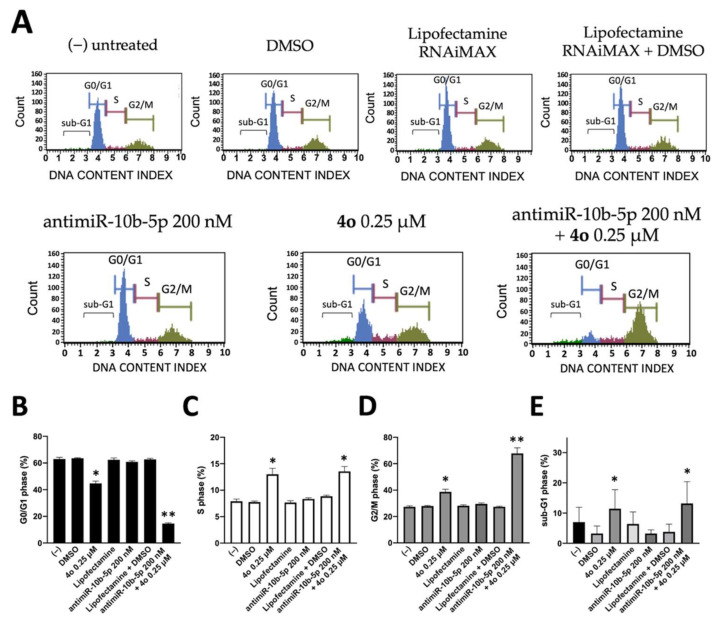
Effects of compound **4o** and antimiR-10b-5p on cell cycle distribution. Representative plot obtained from cell cycle analysis after 72 h treatment with compound **4o** or antimir-10b-5p alone and in combination (**A**) and summary of cells percentage distribution following treatment (**B**–**E**). Results represent mean ± SD (*n* = 3). *p* < 0.05 (*, significant), *p* < 0.01 (**; highly significant).

We first analyzed the effects of compound **4o**, antimiR-10b-5p, or the combined treatment using these molecules on the distribution of the cell cycle. Since molecules exhibiting effects on tubulin assembly might cause alteration of cell cycle parameters, leading to a preferential G2/M blockade, the effects of compounds **4o** on cell cycle distribution were analyzed using flow cytometric analysis and compared to those induced by the antimiR-10b-5p and to those induced by the combined treatment. The cells were cultured for 72 h, as indicated in Figure 5A. Compound **4b** caused an accumulation of cells at the G2/M phase of the cell cycle (from 27.95 ± 0.55% to 38.70 ± 1.91%), with a concomitant decrease in cells in the G0/G1 phase (from 63.55 ± 0.35% to 44.75 ± 1.77%), confirming that compound **4o** impacts cell growth through cell cycle blockade (Figure 5A,B). On the contrary, the antimiR-10b-5p has only minor effects on cell cycle, inducing a very low increase in the percentage of G2/M-phase cells. Interestingly the combined treatment using compound **4o** in the presence of transfection with antimiR-10b-5p caused the highest level of accumulation of the cells into the G2/M phase of the cell cycle (67.8 ± 4.24%, see Figure 5A,B). Furthermore, when we focused on sub-G1 cells, we found the highest values in the combined treatment, suggesting focusing on induction of apoptosis, as sub-G1 peak is a hallmark of apoptosis [54].

### 2.5. Co-Treatment of U251 Cells with Compound ***4o*** and AntimiR-10b-5p: Effects on Apoptosis

**Figure 6:**
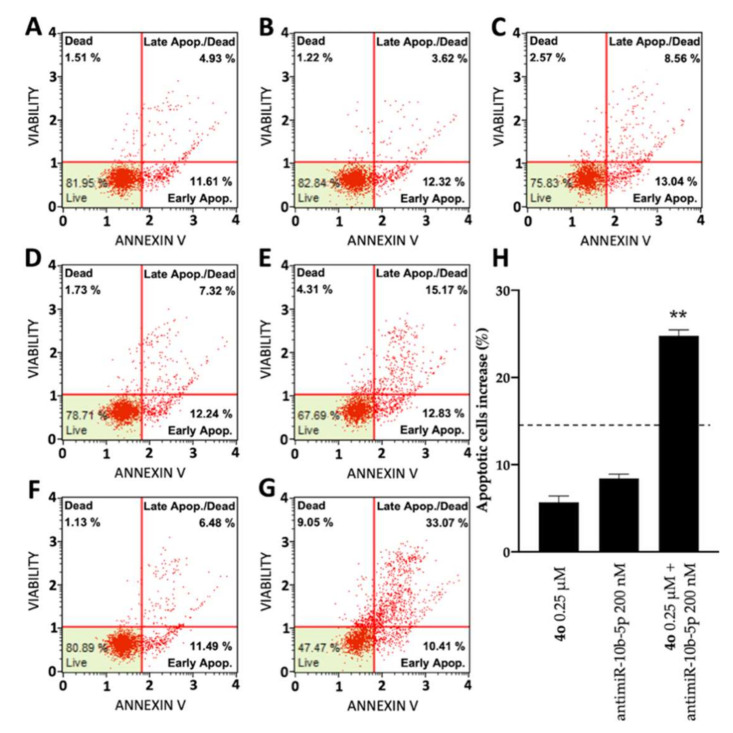
Effects of compound **4o** and antimiR-10b-5p on U251 apoptosis: Annexin V assay. Apoptosis was assayed after 72 h culture. (**A**) Untreated control cells; (**B**) cells treated with DMSO; (**C**) cells treated with 0.25 μM compound **4o**; (**D**) cells treated with lipofectamine; (**E**) cells treated with 200 nM antimiR-10b-5p; (**F**) cells treated with DMSO and lipofectamine; (**G**) cells treated with combined administration of 0.25 μM compound **4o** and 200 nM antimiR-10b-5p. (**H**) summary including results representing % of apoptotic cells of U251 cultures treated as indicated. Results represent mean ± SD (*n* = 3). The dotted line represents the sum of the values obtained with single administrations of the compounds. *p* < 0.01 (**; highly significant).

**Figure 7:**
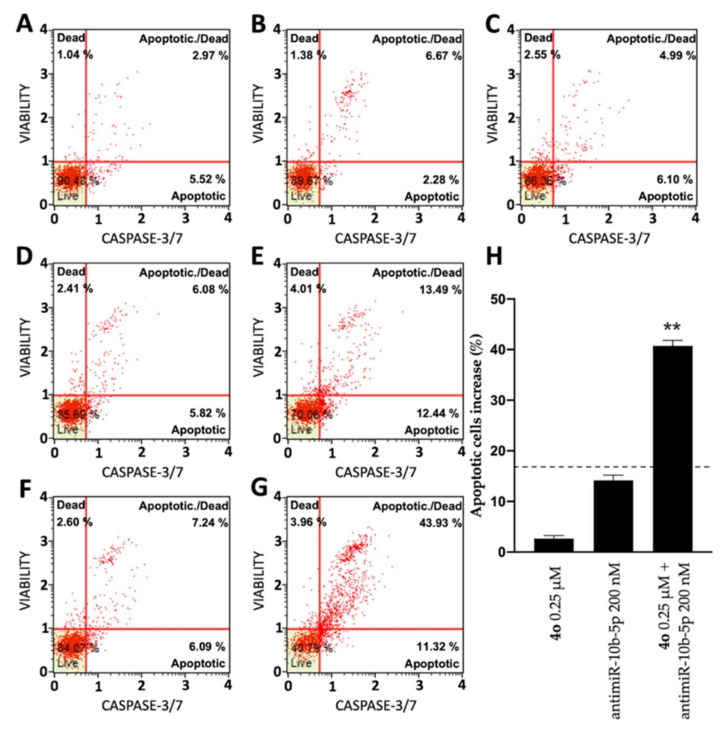
Effects of compound **4o** and antimiR-10b-5p on U251 apoptosis: Caspase 3/7 assay. Apoptosis was assayed after 72 h culture. (**A**) Untreated control cells; (**B**) cells treated with DMSO; (**C**) cells treated with 0.25 μM compound **4o**; (**D**) cells treated with lipofectamine; (**E**) cells treated with 200 nM antimiR-10b-5p; (**F**) cells treated with DMSO and lipofectamine; (**G**) cells treated with combined administration of 0.25 μM compound **4o** and antimiR-10b-5p. (**H**) Summary including results representing % of apoptotic cells of U251 cultures treated as indicated. Results represent mean ± SD (*n* = 3). The dotted line represents the sum of the values obtained with single administrations of the compounds. *p* < 0.01 (**; highly significant).

In order to test the potential induction of apoptosis and cell death of compound **4o** and antimiR-10b-5p administered individually and to verify a possible synergistic effect when administered together, two different apoptosis detection kits were used, the Annexin V assay (Figure 6) and the Caspase 3/7 assay (Figure 7). Figure 6 shows representative Annexin V assay plots demonstrating the effects of **4o** and antimiR-10b-5p administered singularly or together. Both agents induced a low increase of Annexin-V positive cells in comparison with the control after 3 days, but were very effective in inducing apoptotic effects when used in combination. In fact, combined treatments with sub-optimal concentrations of **4o** and antimiR-10b-5p (0.25 μΜ and 200 nΜ, respectively) lead to a sharp induction in apoptosis (24.77 ± 0.67%), a proportion which is much higher than the sum of the effects of singularly added agents (14.12 ± 1.19%; indicated in Figure 6H by the dotted line). It should be noted that the increase in the proportion of apoptotic cells was particularly evident in the “late apoptotic” cell fraction.

The same conclusion derived from the experiments performed using the Annexin V assay can be gathered on the basis of the Caspase-3/7 assay shown in Figure 7. In this case, the induction of apoptosis obtained following combined treatments with sub-optimal concentrations of **4o** and antimiR-10b-5p was 40.75 ± 1.05%, a proportion which is much higher than the sum of the effects of singularly added agents (16.81 ± 0.79%; indicated in Figure 7H by the dotted line).

**Figure 8:**
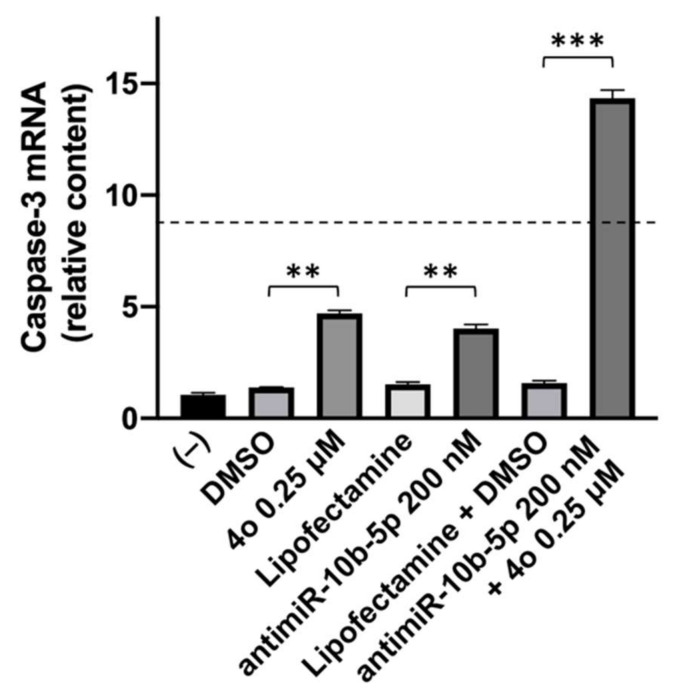
Effects of compound **4o** and antimiR-10b-5p on Caspase-3 mRNA content in treated U251 apoptosis (data normalized on GAPDH). Similar results were obtained using RPL13A and β-actin sequences as internal controls (results shown in Supplementary Material, Figure S7). The dotted line represents the sum of the values obtained with single administrations of the compounds. *p* < 0.01 (**; highly significant), and *p* < 0.001 (***; highly significant).

In order to obtain additional information sustaining an effect on apoptosis, we analyzed by RT-qPCR the effects of the treatments on the expression of Caspase-3 gene, which plays a central role in the execution-phase of cell apoptosis [55]. As shown in Figure 8, 3-day treatment of the cells with compound **4o** or antimR-10b-5p induces an increase of Caspase-3 mRNA. The vehicles, alone or in combinations were ineffective. Interestingly the highest effects on Caspase-3 mRNA induction were obtained in the combined treatment with compound **4o** plus antimR-10b-5p.

## 3. Discussion

Glioblastoma (GBM) patients express high levels of miR-10b-5p, which exerts anti-apoptosis effects and promotes malignant progression [26,27,28,29,30]. The involvement of miR-10b-5p in GBM is supported by the work of Junior et al. [31], who described a high-throughput analysis of the microRNA profile in adult and pediatric primary glioblastomas, demonstrating the role of miR-10b-5p in the tumor aggressiveness. In their study, miR-10b-5p was found to be to most overexpressed microRNAs. In addition, it should be underlined that this microRNA is not expressed in normal brain tissues, normal tissues adjacent to GBM, and a variety of “normal” brain cell lines [27,36,50].

Consistently, inhibition of miR-10b-5p reduced cell proliferation and colony formation in the U251 GBM cell line, firmly establishing that this microRNA acts as an oncogenic miRNA [33,56]. A recent study by Yang et al. confirmed the role of miR-10b-5p in the development of gliomas and suggested that this microRNA could serve as a potential target for the development of new glioma therapies [57]. Fully in agreement, Li et al., using a wound healing and Transwell assays, demonstrated that a miR-10b-5p inhibitor reduced the ability of glioma cells to migrate and invade [56], providing strong evidence that miR-10b-5p inhibition may provide a novel bio-targeting approach for glioma/glioblastoma personalized therapy.

On the other hand, compounds interfering with the microtubule-tubulin equilibrium in glioblastoma cells demonstrated to retain very strong antiproliferative activity, suggesting that compounds targeting tubulin are of great interest for the treatment of GBM [41,47,49]. For this reason, several clinical trials on GBM patients have been designed using tubulin inhibitors such as patupilone (NCT00715013), 2-methoxyestradiol (Panzem: NCT00481455), mebendazole (NCT02644291 and NCT01837862).

In this context, in a recent paper, some of us have described a novel series of tubulin polymerization inhibitors based on the 1-(3′,4′,5′-trimethoxyphenyl)-2-aryl-1*H*-imidazole scaffold [48]. The most active compound in the series (**4o**) exhibited antitumor properties, both in vitro and in vivo [48].

The most important conclusion of the present study is that compound **4o**, when used in combination with a miRNA inhibitor molecule targeting the oncomiRNA miR-10b-5p (antimiR-10b-5p), generates synergistic effects in inducing apoptosis, using the GBM U251 cell line as a model system.

It should be underlined that combined treatments appear of great interest in the development of anticancer approaches [7,8,9,10,11,12], since they are expected to obtain the same biological or therapeutic effect using lower concentrations of two or more drugs, thereby limiting side effects [58]. Importantly, combined therapy might be of great importance in the management of Glioblastoma (GBM), a lethal malignant tumor needing novel therapeutic options. This is mainly due to the fact that, at present, there is no effective pharmacological approach in the treatment of glioblastoma. The first-line drug currently used is Temozolomide, but it is able to extend the life expectancy of patients with GBM by an average of a few months, moreover, very often, this type of tumor is or becomes resistant to this chemotherapeutic agent [4,5,6].

We have already published two studies on synergistic effects of miRNA inhibitors on GBM cell lines when they are co-administered with anticancer agents [13,19]. In the first study, a peptide-nucleic acid (PNA) targeting miR-221-3p was co-administered with a tubulin inhibitor different from that employed in the present study [19]. In the second, a PNA targeting miR-15b-5p was co-administered with sulforaphane [13]. The reason behind studying different miRNA inhibitors combined with different anti-GBM agents is due to the person-to-person variability of response to chemotherapy on the one hand and of onco-miRNAs upregulation on the other. Therefore, one way to explore possible personalized treatments of GBM is to validate several anti-GBM drugs in combination with several anti-miRNA molecules. The reason for moving from the use of PNAs to the use of DNA-analogues targeting miRNAs is related to the fact that, to our knowledge, no anti-miRNA PNAs have reached clinical trials, while several DNA based anti-miRNA molecules are currently under investigation in several clinical protocols on cancer patients. For instance, the clinical trial NCT01849952 has been designed to test the hypothesis that in primary glioma samples, mir-10b expression patterns will serve as a prognostic and diagnostic marker. Furthermore, considering the critical function of antimiR-10b in blocking established glioblastoma growth, the investigators planned to test in vitro the sensitivity of individual primary tumors to antimiR-10b treatment.

In conclusion, our results support the concept that combined treatment of GBM cells with molecules targeting specifically upregulated “oncomiRNA” (in this study, miR-10b-5p) and anticancer agent (in this study, the antitubulin agent **4o**) is a promising strategy in the field of developing effective anti-GBM therapeutic approaches. This strategy may prove of great interest in the personalized approach of precision anti-cancer medicine, since it is well established a patient-to-patient variability in drug response and microRNA pattern.

## 4. Materials and Methods

### 4.1. Chemistry and Reagents

The name of the compound employed in the present study, 2-(3′-chloro-4′-ethoxyphenyl)-1-(3′,4′,5′-trimethoxyphenyl)-1*H*-imidazole (**4o**), was maintained in agreement with the published work by Romagnoli et al. [48] and resuspended in DMSO (see the chemical structure in Figure 1A). The synthesis of **4o** has been described in detail elsewhere [48]. For all cell cultures, RPMI medium supplemented with 10% FBS and 100 mg/mL streptomycin and 100 IU/mL penicillin were employed. RPMI 1640 with L-Glutamine medium (cat.no. FA30WL0500500) was purchased from Carlo Erba Reagents (Cornaredo, Milan, Italy), Trypsin-EDTA solution (cat.no. ECM0920D) from EuroClone (Pero, Milan, Italy), streptomycin and penicillin (cat. no. 11074440001) from Sigma-Aldrich (St. Louis, MA, USA) (Merck KGaA, Darmstadt, Germany), and FBS (cat.no. S1400) from Biowest (Nuaillé, France). For flow cytometry assays, Muse^®^ Annexin-V & Dead Cell kit (cat.no. MCH100105), Muse^®^ Caspase-3/7 kit (cat.no. MCH100108) and Muse^®^ Cell Cycle kit (cat. no. MCH100106) were purchased from Luminex Corporation (Austin, TX, USA). The commercial miRNA inhibitor molecule employed was acquired from Integrated DNA Technologies (IDT, Castenaso, Italy) and specifically synthesized to have high binding efficiency to the target miRNA and protection from endo/exonucleases thanks to 2′OMe and ZEN modification. For antimiR transfection, Lipofectamine RNAiMAX (cat.no. 13778075) and Opti-MEM serum free medium (cat.no. 31985070) were purchased from ThermoFisher Scientific (Waltham, MA, USA), while DMSO (cat. no. D2650) used to resuspend compound **4o**, TRI Reagent (cat. no T9424) used for RNA extraction and MTT powder (cat. no. M5655) used for MTT assay were purchased from Sigma-Aldrich (Merck KGaA).

### 4.2. Cell Lines, Cell Growth Conditions, Antiproliferative Assay

The human glioma U251 cell line was employed [59]. For the antiproliferative test, 8 × 10^4^ cells were seeded in a 12 well plate in a final volume of 500 μL of medium, after 4 h, the cells were treated with tested compounds. The cells were incubated for another 72 h at 37 °C in a humidified 5% CO2 atmosphere. Following 72 h of incubation, cells were detached from the plate by trypsinization and counted using a BECKMAN COULTER^®^ Z2 cell counter (Beckman, Pasadena, CA, USA). The IC50 value (50% inhibitory concentration) is defined as the concentration of a compound that inhibited cell proliferation by 50% (43). The IC50 values presented (± standard deviation) are average values derived from three independent experiments.

### 4.3. Morphological Analysis

Following each singular and combined treatment, the cells were observed, and representative images were acquired using a Nikon Eclipse 80i microscope (Nikon Corporation, Tokyo, Japan) in order to observe whether any morphological changes occurred in the cells following treatment.

### 4.4. AntimiRNA Transfection

For antimiRNA transfection, Lipofectamine RNAiMAX was employed following the manufacturer’s protocol. Briefly, 200 nM antimiR-10b-5p was diluted in 50 μL of serum-free medium (Opti-MEM in this case), and then mixed and incubated 5 min at room temperature with Lipofectamine RNAiMAX diluted in an equal volume of Opti-MEM. At the end of the incubation, cells were treated following the same plating protocol described above. We also added the same amount of Opti-MEM medium in every well of the plate, and we introduced the appropriate vehicle controls for singular and combined treatment (DMSO only, Lipofectamine only, or both).

### 4.5. RNA Extraction

Cells were detached by trypsinization (cat.no. 59428C), collected by centrifugation, and lysed with Tri-Reagent (cat.no. 93289) according to the manufacturer’s instructions. The isolated RNA was washed once with cold 75% ethanol and stored at −80 °C until use. Obtained RNA was dried and dissolved in nuclease-free water before use [13,18] and quantified using a SmartSpec^TM^ Plus Spectrophotometer (Bio-Rad, Hercules, CA, USA) before proceeding with reverse transcription reaction.

### 4.6. Quantitative Analyses of miRNAs

MicroRNA levels were assayed using TaqMan MicroRNA Reverse Transcription Kit (cat.no. 43-665-96) with RT-qPCR and miRNA-specific primers and probes (listed in Table 1) from Applied Biosystems. All samples were run in duplicate using TaqMan Universal PCR Master Mix, no AmpErase UNG 2X (cat.no 4324018) and the CFX96 Touch Real-Time PCR Detection System (BioRad, Hercules, CA, USA). For PCR reactions, the following protocol was employed: 95 °C for 10 min, 95 °C for 15 s, followed by a step at 60 °C for 1 min (last two steps repeated for 50 cycles). Data were collected and analyzed using Bio-Rad CFX Manager Software (Bio-Rad, Hercules, CA, USA). The relative gene expression was calculated using 2^−ΔΔCt^ method, and data normalization was performed using U6 snRNA and hsa-let-7c as reference [18,19].

### 4.7. Analysis of Caspase-3 mRNA by RT-qPCR

The expression of the Caspase-3 gene was verified by RT-qPCR, as described elsewhere [13]. Total RNA was reverse transcribed using random hexamers and TaqMan Reverse Transcription PCR Kit (Thermo Fischer Scientific). Primers and probes used for Caspase-3 detection were purchased from IDT (Integrated DNA Technologies, Castenaso, Italy). The relative mRNA content was calculated using the comparative cycle threshold method and fold change was calculated as 2^−ΔΔCT^. The internal reference sequences employed for normalization were GAPDH, RPL13a and β-actin (primers and probes purchased from IDT).

### 4.8. Effects on the Cell Cycle

The cells were seeded and, after 4 to 5 h, treated with compound **4o** and the anti-miR-10b-5p individually and in combination. Following 72 h of incubation at 37 °C, the cells were detached by trypsinization, washed once in PBS, and fixed with cold 70% EtOH. After 24 h of incubation at −20 °C, fixed cells were washed in PBS and resuspended in 200 µL of Muse*^®^* Cell Cycle Reagent and incubated for 30 min at room temperature protected from light. Finally, the cell suspension was transferred into a new tube and the samples analyzed by flow cytometry using Guava*^®^* Muse*^®^* Cell Analyzer (Luminex Corp., Austin, TX, USA) [19].

### 4.9. Cell Apoptosis Assays

Apoptosis assays were performed with Guava^®^ Muse^®^ Cell Analyzer instrument, and its relative kits according to the instructions supplied by the manufacturer. After 72h of treatment, supernatant was collected and cells were washed with sterile PBS, trypsinized, and resuspended in RPMI medium supplemented with 10% FBS together with respective supernatants. Finally, 100 μL of cell suspension were incubated with 100 μL Muse^®^ Annexin V & Dead Cell reagent at room temperature and protected from light for 20 min. Samples were then analyzed using Guava^®^ Muse^®^ Cell Analyzer (Luminex Corp.) and data acquired utilizing the Annexin V and Dead Cell Software Module (Luminex Corp.) [13,60]. For the analysis of Caspase-3/7 activity following treatments, the Muse^®^ Caspase-3/7 Kit was employed. After the treatment, trypsinization was performed, and 50 μL of cell suspension was incubated with 5 μL of caspase 3/7 reagent for 30 min (under strict protection from light). After 25 min of incubation, 150 μL of 7-AAD working solution was added to each tube and incubated for 5 min before reading the samples. Samples were then analyzed using Guava^®^ Muse^®^ Cell Analyzer (Luminex Corp.) and data acquired utilizing the Caspase-3/7 Software Module (Luminex Corp.) [19,60].

### 4.10. Statistics

Results are expressed as mean ± standard error of the mean (SEM). Comparisons between groups were made by using paired Student’s *t*-test using Graph Pad Prism 9 software or ANOVA followed by Dunnet multiple comparison test. Statistical significance was defined with *p* < 0.05 (*, significant), *p* < 0.01 (**; highly significant), and *p* < 0.001 (***; highly significant).

## Figures and Tables

**Table 1:** List of assays employed for miRNA detection.

miRNA Name	Assay ID (Applied Biosystems by Thermo Fisher Scientific, Inc., Waltham, MA, USA)
hsa-miR-10b-5p	002218
hsa-U6 snRNA	001973
hsa-let-7c-5p	000379

## Data Availability

The datasets and materials generated and/or analyzed during the present study are available from the corresponding author upon reasonable request.

## Supplementary Materials

The following supporting information can be downloaded at: https://www.mdpi.com/article/10.3390/ijms23115991/s1.
