# Supplementary material for: Synergistic Effects of A Combined Treatment of Glioblastoma U251 Cells with An Anti-miR-10b-5p Molecule and An AntiCancer Agent Based on 1-(3′,4′,5′-Trimethoxyphenyl)-2-Aryl-1H-Imidazole Scaffold
Source: Int J Mol Sci. 2022 May 26;23(11):5991. doi: 10.3390/ijms23115991 (PMC9181338; doi:10.3390/ijms23115991)
Supplement: Supplementary file 1 [file ijms-23-05991-s001.zip › ijms-1686488-supplementary-1.pdf]

International Journal of
Molecular Sciences

MDPI

Article

Synergistic effects of a combined treatment of glioblastoma U251 cells with an anti-miR-10b-5p molecule and an anticancer agent based on 1-(3′,4′,5′-trimethoxyphenyl)-2-aryl-1*H*-imidazole scaffold

Matteo Zurlo [1], Romeo Romagnoli [2], Paola Oliva [2], Jessica Gasparello [1], Alessia Finotti [1,*] and Roberto Gambari [1,*]

[1] Department of Life Sciences and Biotechnology, Ferrara University, 44121 Ferrara, Italy; M.Z., matteo.zurlo@unife.it; J.G., jessica.gasparello@unife.it; A.F., alessia.finotti@unife.it; R.G., gam@unife.it
[2] Department of Chemical, Pharmaceutical and Agricultural Sciences, Ferrara University, 44121 Ferrara, Italy; R.R, rmr@unife.it; P.O., lvopla@unife.it
* Correspondence: Correspondence: R.G., gam@unife.it; A.F., alessia.finotti@unife.it.

Keywords: microRNA; antimir; miR-10b-5p; glioma; apoptosis; tubulin; 1-(3′,4′,5′-trimethoxyphenyl)-2-aryl-1*H*-imidazole; combination therapy

Supplementary Material

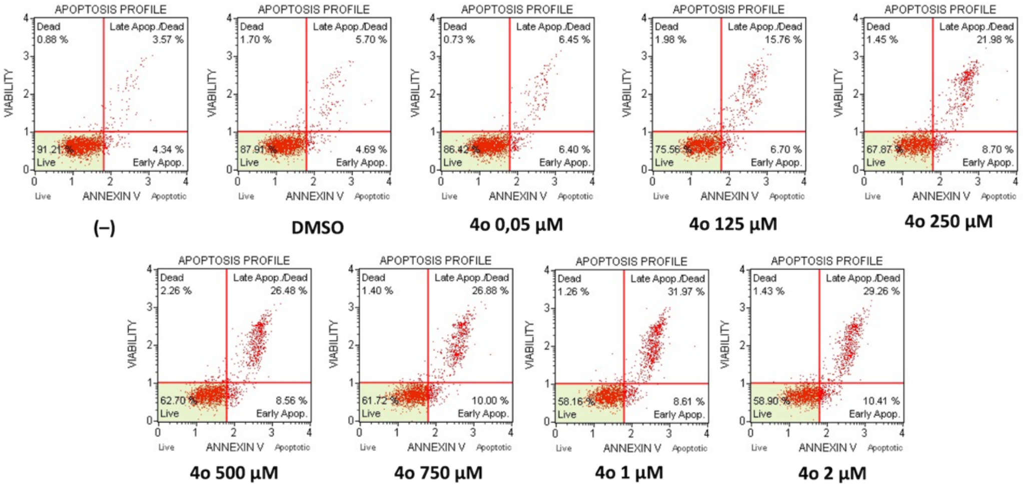

Figure S1. Effects of compound 4o on apoptosis of U251 cells. The annexin V assay was performed on U251 cells treated for 3 days in the presence of the indicated concentrations of 4o.

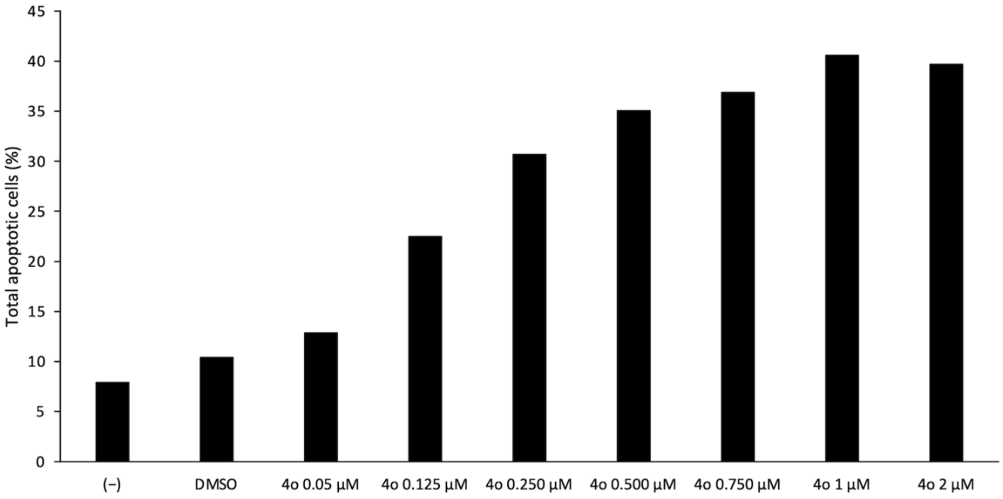

Figure S2. Summary representing the mean increase of % of apoptotic cells in U251 cells treated with increasing concentrations of compound 4o.

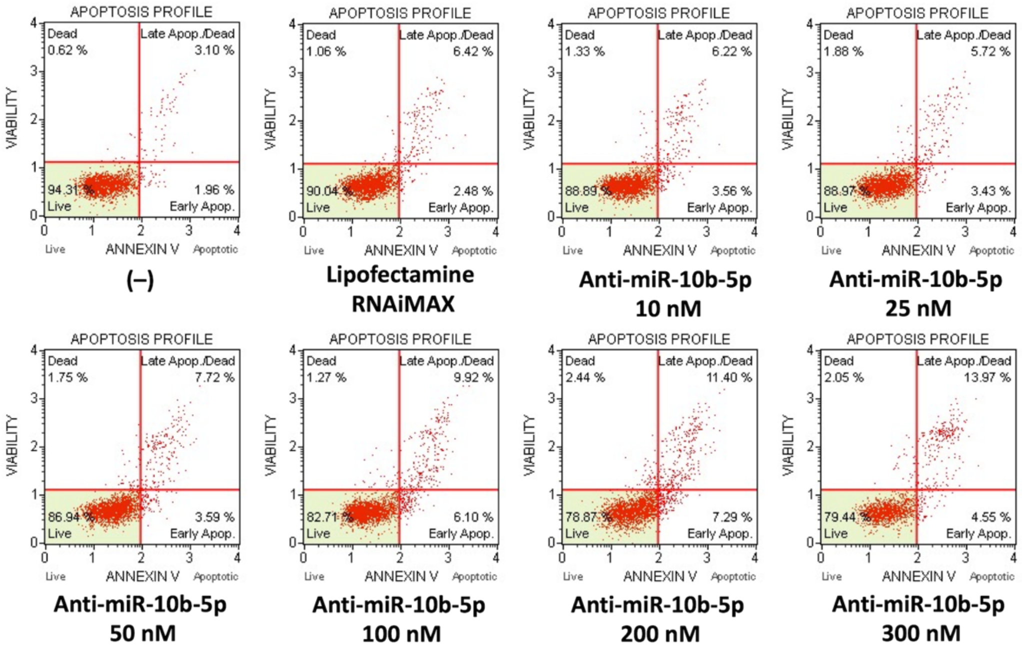

Figure S3. Effects of anti-miR-10b-5p on apoptosis. Annexin V assay plots performed on untreated U251 cells (-) or cells cultured for 3 days in the presence of DMSO or the indicated concentrations of anti-miR-10b-5p. These data generated the summary results presented in Figure 3F.

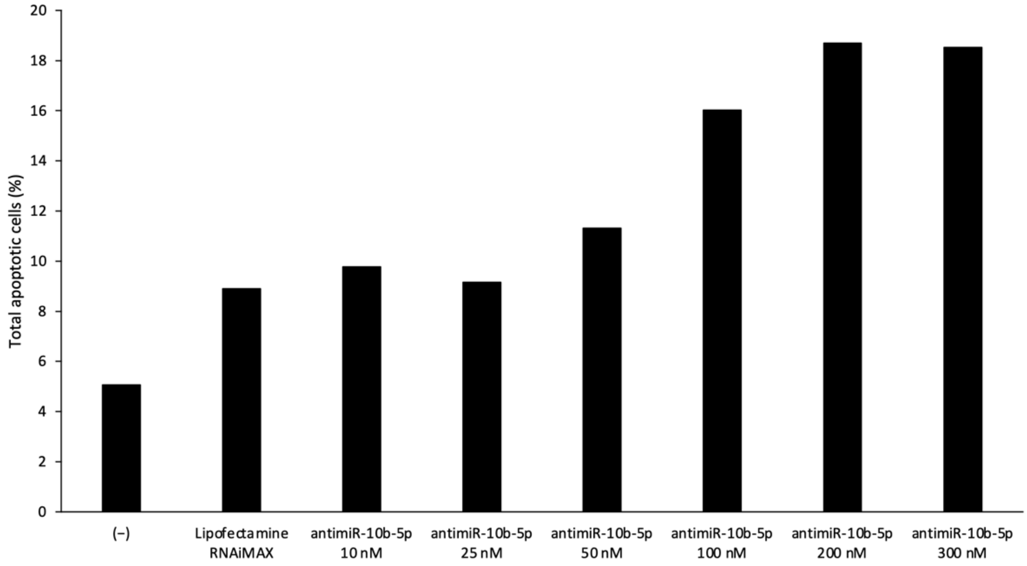

Figure S4. Summary representing the mean increase of % of apoptotic cells in U251 cells treated with increasing concentrations of antimiR-10b-5p.

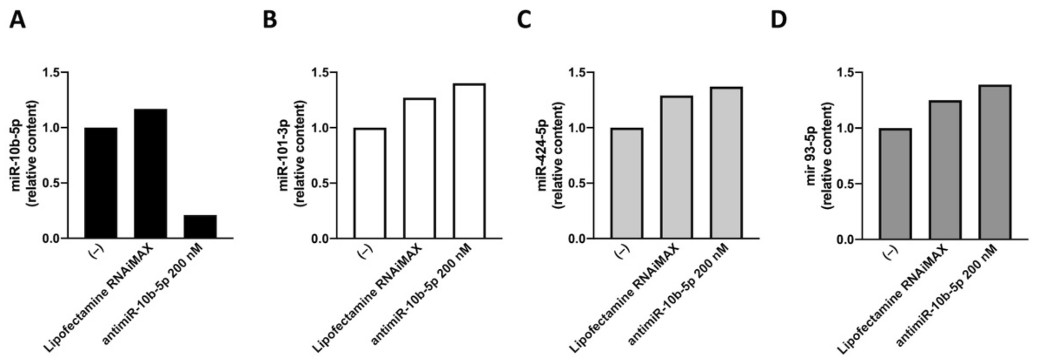

Figure S5. Effects of the treatment of U251 cells with anti-miR-10b-5p on the expression different miRNAs. U251 cells were either untreated (-) or treated for 3 days with lipofectamine RNAiMAX or with anti-miR-10b-5p, as indicated. RT-qPCR was performed to quantify the expression of miR-10b-5p (A), miR-101-3p (B), miR-424-5p (C) and miR-93-5p (D). Let-7c was used as internal control.

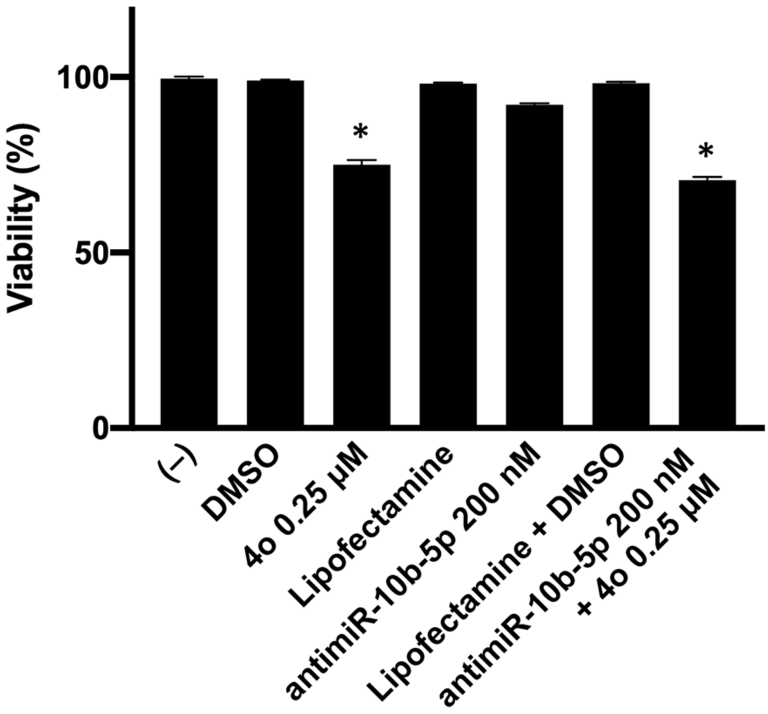

Figure S6. Cytotoxic effects of compound 4o and pre-miR-10b-5p on U251 cells. U251 cells were treated as indicated for 3 days and effects on viability were quantified by MTT assay.

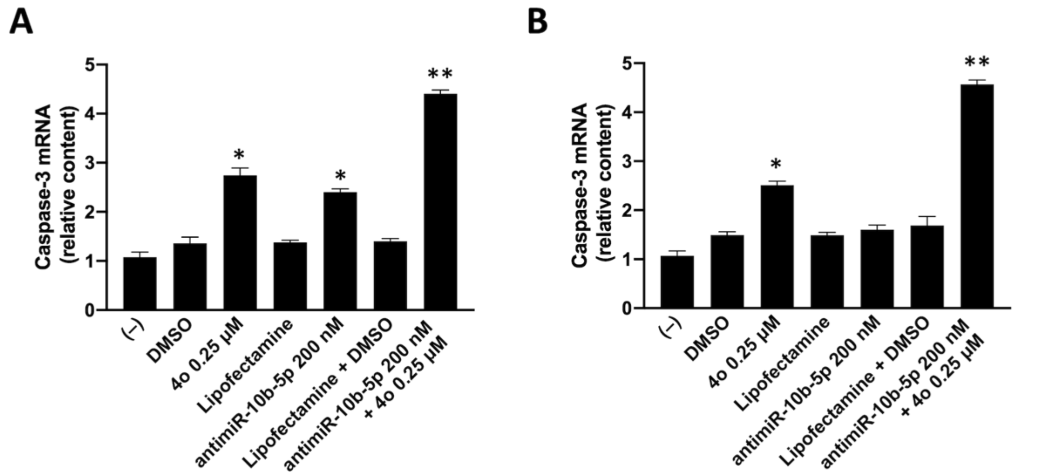

Figure S7. Effects of compound 4o and antimiR-10b-5p on Caspase-3 mRNA content in treated U251 apoptosis. U251 cells have been cultured as indicated for 3 days. Then RNA was extracted and Caspase-3 was analyzed by RT-qPCR using RPL13a (A) and β-actin (B) sequences as internal controls.

Supplementary methods

MTT assay

Cytotoxicity of tested compound was assessed by MTT assay. Briefly, cells were seeded in a 96-multi-well plate at a density of 8×10^3 cells/well and after 4 h, treated with compound 4o (0,25 µM) and antimir-10b-5p (200 nM) individually and in combination. Cells were incubated at 37°C for a further 72 h and at the end of the incubation period, MTT was added to each well at a final concentration of 0.5 mg/ml. Following 5 h of incubation at 37°C, the medium was discarded, and dimethyl sulfoxide (DMSO) was added; the plate was stirred for 30 min prior to acquisition of data. The absorbance was measured at 570 nm using the SUNRISE microplate reader (Tecan Group, Ltd., Mannedorf, Switzerland).

RT-qPCR

Additional PCR data on tumor suppressor miRNAs (101-3p, 424-5p, 93-5p) were obtained using specific PCR assays listed in supplementary Table S1, let-7c was used as reference for normalization. Reverse transcription protocol and general PCR protocol was the same described in methods section.

miRNA name	Assay ID (Applied Biosystems by Thermo Fisher Scientific, Inc., **Waltham, MA, USA**)
hsa-miR-10b-5p	002218
hsa-let-7c-5p	000379
hsa-miR-101-3p	002253
hsa-miR-424-5p	000604
hsa-miR-93-5p	001090

Table S1. List of assays employed for miRNA detection.
